# Differences in somatic mutation landscape of hepatocellular carcinoma in Asian American and European American populations

**DOI:** 10.18632/oncotarget.9636

**Published:** 2016-05-26

**Authors:** Song Yao, Christopher Johnson, Qiang Hu, Li Yan, Biao Liu, Christine B. Ambrosone, Jianmin Wang, Song Liu

**Affiliations:** ^1^ Department of Cancer Prevention and Control, Roswell Park Cancer Institute, Buffalo, NY, USA; ^2^ Department of Biostatistics and Bioinformatics, Roswell Park Cancer Institute, Buffalo, NY, USA

**Keywords:** somatic mutation, hepatocellular carcinoma, disparity, TCGA, ancestry

## Abstract

The incidence rate of hepatocellular carcinoma (HCC) is higher in populations of Asian ancestry than European ancestry (EA). We sought to investigate HCC mutational differences between the two populations, which may reflect differences in the prevalence of etiological factors. We compared HCC somatic mutations in patients of self-reported Asian American and EA from The Cancer Genome Atlas (TCGA), and assessed associations of tumor mutations with established HCC risk factors. Although the average mutation burden was similar, *TP53* and *RB1* were mutated at a much higher frequency in Asian Americans than in EAs (*TP53*: 43% vs. 21%; *RB1*: 19% vs. 2%). Three putative oncogenic genes, including *TRPM3*, *SAGE1*, and *ADAMTS7*, were mutated exclusively in Asians. In addition, VEGF binding pathway, a druggable target by tyrosine kinase inhibitors such as sorafenib, was mutated at a higher frequency among Asians (13% vs. 2%); while the negative regulation of IL17 production, involved in inflammation and autoimmunity, was mutated only in EAs (12% vs. 0). Accounting for HCC risk factors had little impact on any of the mutational differences. In conclusion, we demonstrated here mutational differences in important cancer genes and pathways between Asian and European ancestries. These differences may have implications for the prevention and treatment of HCC.

## INTRODUCTION

Liver cancer is the seventh most common type of cancer and the third deadliest cancer globally, with approximately 782,000 new cases and 746,000 deaths in 2012 [1]. Hepatocellular carcinoma (HCC) is the dominant histological type of liver cancer. In the U.S., the incidence of HCC tripled between 1975 and 2005 [2]. There are persistent disparities in HCC incidence by geographical area and ancestry. Some Asian and African countries have very high rates, with the highest found in Mongolian men at 116.6 per 100,000 person-years, in contrast to 3.8 per 100,000 person-years in Northern Europe [3]. Ancestral differences also exist within the U.S. such that Asian Americans have higher incidence rates than EAs [2].

HCC ethnic disparities have largely been attributed to a high prevalence of Hepatitis B virus (HBV) infection among Asians [4, 5]. In addition, dietary aflatoxin B1 exposure is another common risk factor for HCC in some developing Asian countries, whereas Hepatitis C virus (HCV) infection, chronic alcohol abuse, and metabolic syndrome are common risk factors in Western countries [6]. The differences in distributions of risk factors for HCC across countries may not only lead to an unequal cancer incidence rate, but also distinct tumor biology. However, the contributions of differential exposure to HCC risk factors to ancestral differences in tumor biology are unclear.

HCC may be an appropriate cancer type to study ancestral differences in tumor biology and exposures because a majority of HCCs can be attributed to discrete risk factors, whereas etiological causes are still somewhat ambiguous for many other cancers. With the advent of next-generation sequencing, several recent studies have profiled the genomic landscape of HCC [7–13], and identified a number of significantly mutated genes (SMGs). In the meantime, it has become increasingly clear that exogenous and endogenous exposures leave characteristic fingerprints on tumor genomes, which can be deciphered by mutational patterns [14]. This makes it possible to investigate etiological links between cancer risk factors and somatic mutations, as demonstrated in a recent study by Schulze et al. [13]. In European HCC patients, alcohol was associated with mutations in *CTNNB1*, *TERT*, *CDKN2A*, *SMARCA2*, and *HGF*, and HBV infection was associated with mutations in *TP53*.

These advances have now made it feasible to compare tumor mutations by ancestry and, further, to relate the differences to etiological risk factors. This may address the question of whether tumor biological differences by ancestry truly exist. Moreover, it may also provide important information regarding the role of ancestry in cancer treatment. For example, in non-small cell lung adenocarcinoma, the efficacy of tyrosine kinase inhibitors, including gefitinib and erlotinib, is linked to epidermal growth factor receptor (*EGFR*) mutations, which are much more prevalent among Asian patients than EAs (30% vs. 7%) [15–17]. This may explain the enhanced sensitivity and clinical response seen in Asian patients treated with these drugs. Interestingly, sorafenib, a vascular endothelial growth factor (VEGF) receptor kinase inhibitor approved for advanced HCC, also shows differences in clinical response between Asian and EA patients [18, 19]. It is unclear whether mutations in the VEGF pathway contribute to these differential treatment responses.

To date, published HCC sequencing studies were often conducted in Asians or EAs separately [7–13, 20]. The only trans-ancestry study by Totoki et al analyzed ancestry-dependent diversity in HCC mutation signature as aggregated patterns of all nucleotide changes, but not at the depth of cancer genes or pathways [21]. By leveraging the whole-exome sequencing data from The Cancer Genome Atlas (TCGA), we compared somatic mutations at the levels of genes and pathways between the two populations, and further, examined whether the differences were attributed to known risk factors.

## RESULTS

### Asian American and European American HCC patients in TCGA

Data from 54 Asian American and 104 EA HCC patients who self-identified as non-Hispanic ethnicity and had both exome sequencing data and clinical information were obtained from TCGA at the time of the study (March 2015). The descriptive characteristics of the patient population are shown in Supplementary Table 1. Asian American cases were younger, more likely to be male, and without a family history of cancer. No significant difference in mutation burden was found between the two populations, with an average number of 139 (range: 31-359) mutations in Asians and 124 (range: 2-445) in EAs, p-value=0.49 (Supplementary Figure 1).

### Genes differentially mutated in HCC tumors from Asian Americans and European Americans

**Figure 1:**
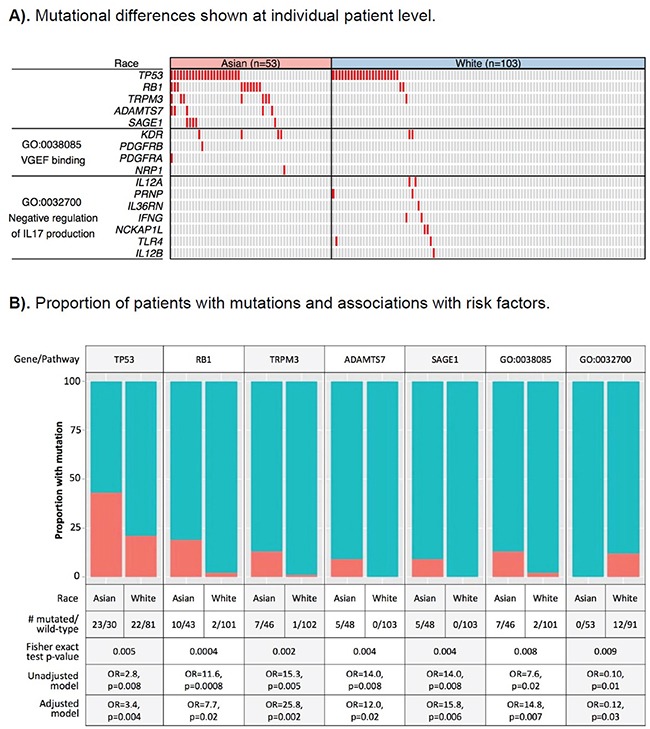
Differentially mutated genes and pathways between hepatocellular carcinoma (HCC) patients of Asian and European ancestry **A.** Differentially mutated genes and pathways are plotted by row and tumors by column. A tumor with a mutation is depicted in red. **B.** Red color: the proportion of tumors with mutation; blue color: the proportion of tumor with wild-type gene/pathway; GO:0038085, vascular endothelial growth factor (VEGF) binding pathway; GO:0032700, negative regulation of interleukin 17 (IL17) production. OR: odds ratio derived from exact logistic regression models, with the odds of the gene/pathways being mutated vs. wild-type and independent variables being ethnicity (patients of Asian vs. European ancestry). In the adjusted models, HCC risk factors (HBV, HCV, chronic liver disease, alcohol), as well as age at diagnosis, gender, and family history of cancer were included as covariates.

We identified five genes differentially mutated between the two ancestral groups at a nominal p-value <0.01 (Figure 1A Complete mutation data used to generate the figure with functional annotation is provided in Supplementary Table 2). To address the issue of unbalanced sample size of Asian Americans and EAs in TCGA and multiple testing, we subsequently validated the initial findings using a permutation-based test (data not shown). Among the five genes, *TP53* and *RB1* are known driver genes in HCC. *TP53* was mutated in 43% of Asian patients, more than twice that in EAs (21%). The difference in mutation rate of *RB1* was even larger, with 19% in Asians and only 2% in EAs. Except for *TP53* and *RB1*, other known “driver” genes HCC from previous studies, including *CTNNB1*, were not differentially mutated by ancestry (Supplementary Table 3). The other three differentially mutated genes, including *SAGE1*, *TRPM3*, and *ADAMTS7*, have not been implicated in HCC, but in some other cancers [22–25].

### Pathways/biological processes differentially altered in HCCs from Asian Americans and EAs

**Figure 2:**
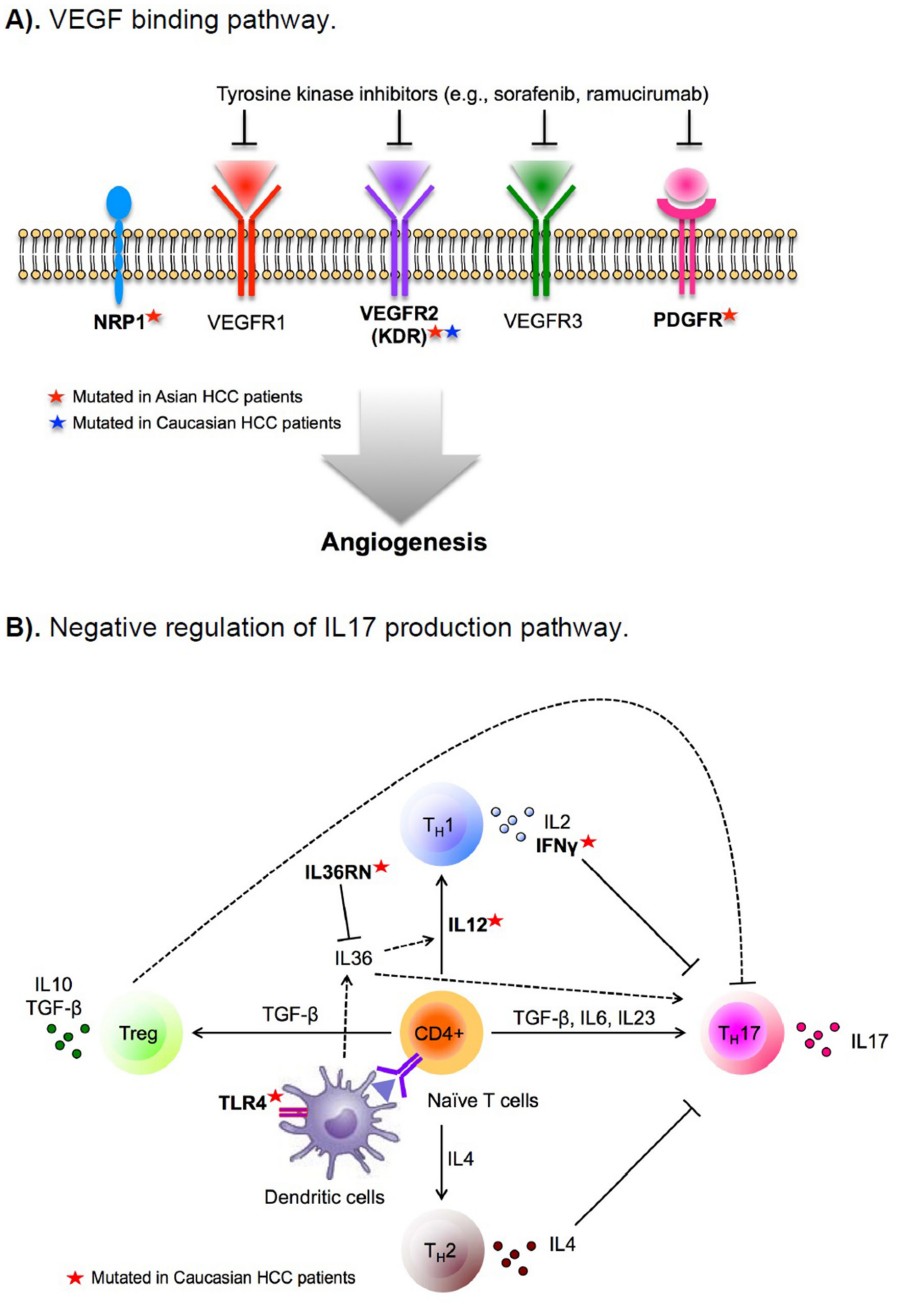
Differentially altered functional pathways in hepatocellular carcinoma (HCC) from patients of Asian and European ancestry **A.** VEGF binding pathway. **B.** Negative regulation of IL17 production pathway.

As shown in Supplementary Table 4, a number of the top gene ontology (GO) pathways/biological processes that were altered at a higher frequency in Asian Americans than in EAs were driven by *TP53* and *RB1* mutations, as already identified in the above gene-level analysis. An additional pathway more likely to be altered in Asian American patients was the VEGF binding pathway (GO:003270, 13% in Asians vs. 2% in EAs, p-value=0.008) (Figure 1A), which is well known for its role in angiogenesis and metastasis. *KDR*, which encodes VEGF receptor 2, was mutated in 7.5% of Asian patients, compared to 1.9% in EAs. The other three genes in the pathway, including *PDGFRA*, *PDGFRB*, and *NRP1*, were mutated only in Asians. The former two genes encode palate-derived growth factor (PDGR), a complementary angiogenic factor to VEGF; *NRP1* encodes neuropillin, a co-receptor of VEGF (Figure 2A). Based on the Drug Gene Interaction Database (DGIbd) [26, 27], all four genes were potentially druggable; plus, all but *NRP1* were also clinically actionable and can be targeted by receptor tyrosine kinase inhibitors, such as sorafenib. KDR can also be targeted by its specific inhibitor, ramucirumab.

We also identified several pathways/biological processes more likely to be altered in tumors from EA patients (Supplementary Table 4). The most notable pathway was the negative regulation of interleukin 17 (IL17) production (GO:0038085), where seven genes involved in T-cell and immunocyte activation through interferon gamma harbored mutations in 12% of EA patients, but none in Asian American patients (p-value=0.009) (Figure 2B). IL17, produced by T-helper 17 cells, has been shown to contribute to chronic inflammation of the liver and autoimmunity, and may play a role in liver tumorigenesis [28, 29]. The most notable mutated genes in this pathway included *IFNG*, *IL12A* and *IL12B* and encoding interferon gamma and IL12, respectively, as well as *TLR4* encoding toll-like receptor 4, a critical regulator of IL17-mediated inflammation [30, 31].

### Associations of HCC risk factors with mutated genes and pathways

**Figure 3:**
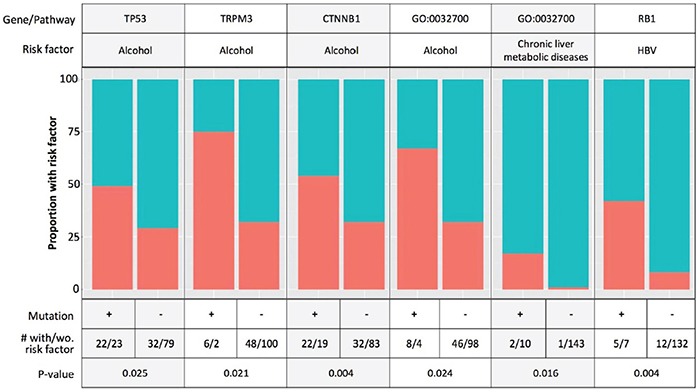
Associations of known hepatocellular carcinoma (HCC) risk factors with mutated genes and pathways Red color: the proportion of patients with exposure to the risk factor; blue color: the proportion of patients without exposure to the risk factor; GO: 0038085, vascular endothelial growth factor (VEGF) binding pathway; GO:0032700, negative regulation of interleukin 17 (IL17) production.

We next examined known HCC risk factors, including alcohol use, HBV and HCV infection, and chronic liver metabolic diseases (hemochromatosis, non-alcoholic fatty liver disease) with known HCC SMGs and the differentially mutated genes and pathways by ethnicity. Because data on aflatoxin exposure were not reported in TCGA, we queried the signature *TP53* R249S mutation for aflatoxin exposure [32] and found none present in either Asian American or EA cases. Figure 3 shows the top associations of risk factors and mutated genes and pathways, and the complete results of all associations tested are provided in Supplementary Table 5. Alcohol consumption was associated with mutations in *CTNNB1*, *TP53*, *TRPM3*, as well as the negative regulation of the IL17 production pathway. HBV infection was associated with *RB1* mutations, but not *TP53* mutations as previously reported [13]. Chronic liver diseases were also associated with the negative regulation of the IL17 production pathway. In addition, male gender was associated with mutations in *TP53* and VEGF binding pathway.

### Contribution of risk factors to the observed mutational differences by ethnicity

As shown in Supplementary Table 1, HBV infection was more common in Asian Americans than in EAs (24.5% vs. 3.9%, p-value <0.001), while HCV infection was more common in EAs (5.7% vs. 18.4%, p-value=0.04). Alcohol consumption was similar between the two groups (30.2% in Asians and 35.9% in EAs). Chronic liver diseases were uncommon in the TCGA population, present in only three EAs and in no Asians.

In analyses of the contribution of HCC risk factors to the five differentially mutated genes and two pathways between Asian American and EA patients, controlling for the risk factors, as well as age at diagnosis, gender, and family history of cancer, had no apparent impact on the any of the ethnic differences that were associated with these risk factors, including *TP53*, *RB1*, *TRPM3*, and negative regulation of the IL17 production pathway. The odds ratio associated with ethnicity remained significant after adjusting for the risk factors (Figure 1B).

## DISCUSSION

Liver cancer is a highly lethal disease, with a dismal five-year survival rate below 20% [33]. It puts an unequally heavy burden on some Asian communities, including Asian Americans in the U.S. [2, 3]. In this study, we observed marked differences in tumor mutations in major cancer genes (i.e., *TP53* and *RB1*) and targetable pathways (i.e., VEGF binding pathway), between Asian American and EA HCC patients. Although known HCC risk factors were associated with some of the mutations, they could not explain the observed mutational differences.

We also found several functional pathways differentially altered between Asian and EA HCC patients. Notably, Asian Americans were more likely to harbor mutations in the VEGF binding pathway, which is critical to tumor angiogenesis and progression. Interestingly, Asian patients appeared to benefit less from VEGF inhibitor, sorafenib, than EAs when two separately conducted trials were compared. This difference might be related to differences in somatic mutations [18, 19]. A recent observational study, however, showed no apparent differences in therapy benefits between the two groups when treated with sorafenib at the same institution [34]. The relatively small sample size of Asian patients in the study might render inadequate statistical power for analyzing ethnic differences in benefits from sorafenib. Our finding of a higher frequency of altered VEGF binding pathways in Asian American HCC patients than in EA patients calls for continued efforts to investigate the role of ethnicity in clinical response to sorafenib and other targeted cancer therapies.

Another notable pathway differentially altered between Asian Americans and EAs is the negative regulation of IL17 production. IL17 is produced mainly by T-helper 17 (Th17) cells, which are involved in liver inflammatory processes and autoimmunity, and have been implicated in inflammation-related liver diseases, including HCC [28, 29]. Some studies have reported a negative relationship between tumor-associated IL17 responses and survival of HCC patients [35]. The production of IL17 is suppressed by IFNγ, IL4, and IL12, which favor the production of Th1 or Th2 cells [36]. We found that approximately 12% of HCC tumors from EAs carried mutations in this pathway, but none in Asians. Further, alterations in the negative regulation of IL17 production were associated with high alcohol consumption and chronic liver diseases in our analysis. Because alcohol use was similar between Asian Americans and EAs (30% vs. 36%) and no mutation in this pathway was found in Asians, the association between alcohol and the mutation in this pathway was driven entirely by EAs.

The above findings support the existence of biological differences at the level of somatic mutations between Asian American and EA HCC patients. An apparent explanation for the differences could be known HCC risk factors, since HBV was more prevalent in Asian Americans, while HCV infection was more prevalent in EAs in the study. However, accounting for HBV, HCV and other risk factors had little impact on any of the observed differences in mutation frequency. Possible explanations for this could be that additional risk factors for HCC might have not been accounted for. Alternatively, the associations between HCC risk factors and somatic mutations might differ between Asian Americans and EAs, as in the case of high alcohol consumption and negative regulation of the IL17 production pathway. Either of the two explanations would be rather provocative and may warrant exploration in the future.

Our analyses based on TCGA data had some limitations. First, the sample size, particularly the Asian American population, was relatively small. Second, we focused on point mutations and it would be interesting to extend the analyses to other genetic abnormalities, such as copy number changes. Because we relied on whole-exome sequencing data, mutations in the *TERT* promoter region, which were reported to occur in 54% HCC patients [21], were not captured in the data and not analyzed. Third, TCGA somatic data are not vigorously annotated with risk factors or clinical outcomes. The available data on HCC risk factors were reported by individual institutions submitting samples to TCGA. The heterogeneity in the methods and quality of data collection across institutions might result in exposure misclassification. Particularly, it should be noted that the prevalence of HBV infection among Asian American HCC patients (24.5%) and HCV infection among EA HCC patients (18.4%) in the TCGA dataset appeared to be lower than those reported in other studies in the U.S. [37–39]. Nevertheless, we successfully replicated several previously reported mutation-risk factor associations in the study, which supports the validity of the risk factor data in TCGA. Fourth, it would also be interesting to examine HCC somatic mutations in African-Americans and Hispanics in future studies, the two populations with a rapidly increasing liver cancer incidence in recent decades [40].

In conclusion, we found significant ethnic differences in tumor somatic mutations between Asian American and EA HCC patients, which may not be caused by differential distribution of established HCC risk factors. The findings suggest that there are ethnic differences in the biology of HCC at a genomic level, and ethnicity may have important implications for the prevention and treatment of the disease.

## MATERIALS AND METHODS

### Data access

The TCGA Data Portal was utilized to access HCC data. Only cases with both curated mutation calling (not raw sequencing data) from tumor whole-exome sequencing data and annotated clinical data were included. Cases of self-reported Asian American and European American were included, and those of Hispanic ethnicity were removed.

### Calculation of mutation frequency of single genes

The number of patients with at least one mutation in each gene, as well as the number of mutations per patient was calculated. Two patients displaying the hypermutator phenotype, defined as Q3 + 4.5 times inter-quartile range (IQR) [41], were removed. Mutation frequency for each gene was calculated as the percentage of cases carrying at least one Tier 1 single nucleotide variant (SNV) (coding synonymous, nonsynonymous, splice site, and non-coding RNA variants), and compared between Asian Americans and EAs using Fisher's exact test. To correct for potential bias due to an unbalanced sample size between the two groups (103 vs. 53), a permutation-based test was used by comparing the smaller Asian American group to a sub-sample of the same number randomly drawn from the EA cases. The process was repeated 1,000 times to derive an empirical p-value.

### Analysis of somatic alterations in pathways and biological processes

GO database [42] was used to determine whether any functionally relevant groups of genes were altered at a significantly higher frequency in one group than in the other. Entrez ID for each gene was mapped to GO terms based on the file Gene2GO acquired from NCBI. The frequency of alterations in each GO term was calculated and compared between Asian American and EA cases using Fisher's exact test.

### Analysis of somatic mutations with known HCC risk factors

HCC risk factors for each patient were obtained through the TCGA Data Portal. As aflatoxin exposure was not reported, we queried the mutational signature of aflatoxin exposure as defined by missense R249S (AGG to AGT) on codon 249 of *TP53* [32]. Fisher's exact test was used to determine whether a risk factor had a different distribution between the two ancestral groups and whether a risk factor was associated with a mutated gene or a GO term. To explore whether the observed differences in somatic mutation were attributed to known HCC risk factors and to accommodate small numbers in some categories, exact logistic regression was used to model somatic mutations with inclusion of both ancestry and HCC risk factors (HBV, HCV, chronic liver disease, alcohol) in the full model, as compared to the base model with ancestry only.

## SUPPLEMENTARY FIGURE AND TABLES






