# Supplementary material for: Differences in somatic mutation landscape of hepatocellular carcinoma in Asian American and European American populations
Source: Oncotarget. 2016 May 26;7(26):40491–9. doi: 10.18632/oncotarget.9636 (PMC5130022; doi:10.18632/oncotarget.9636)
Supplement: Supplementary file 1 [file oncotarget-07-40491-s001.pdf]

Differences in somatic mutation landscape of hepatocellular carcinoma in Asian American and European American populations

SUPPLEMENTARY FIGURE AND TABLES

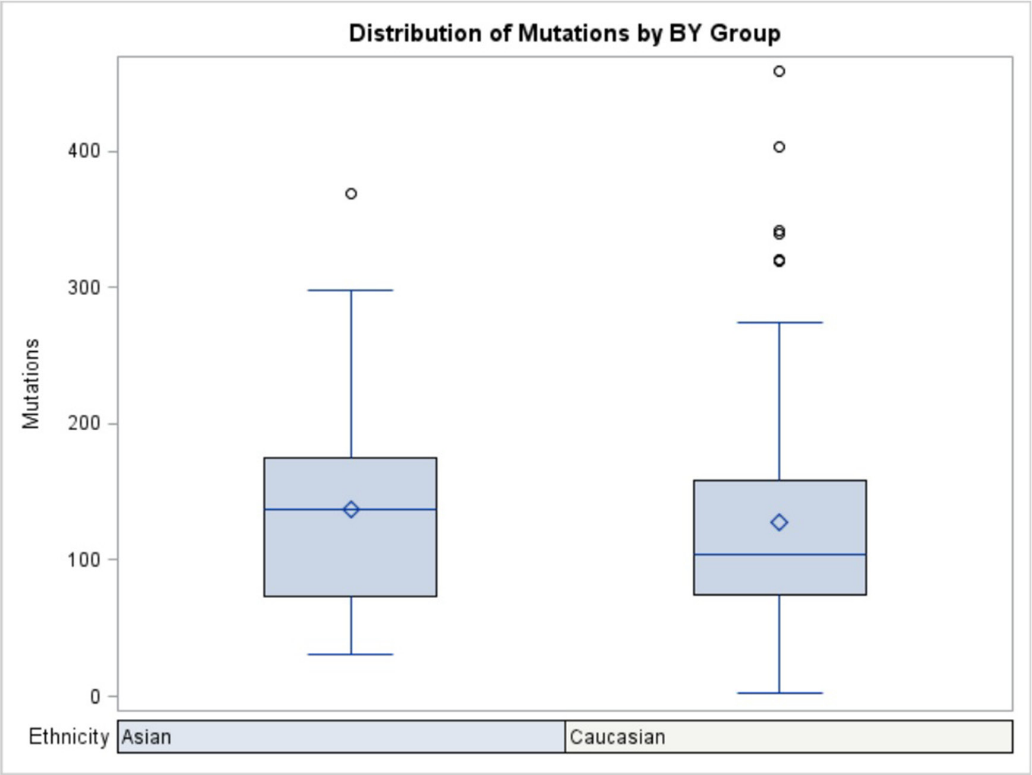

Supplementary Figure S1: Mutation burden of hepatocellular carcinoma in Asians and Caucasians.

Supplementary Table S1: Descriptive characteristics of the TCGA hepatocellular carcinoma patients

	Asian (n=53)	Caucasian (n=103)	P
Age at diagnosis, year, mean (sd)	52.2 (11.9)	64.9 (13.1)	<0.001
Gender, n (%)			0.00971
Male	41 (77.4)	58 (56.3)	
Female	12 (22.6)	45 (43.7)	
Family history of cancer, n (%)			
Yes	6 (11.3)	47 (45.6)	0.014
No	38 (71.7)	37 (35.9)	
Missing	9 (17.0)	19 (18.4)	
Country of origin, n (%)			0.0209
U.S.	8 (15.1)	88 (85.4)	
Canada	8 (15.1)	12 (11.7)	
Vietnam	25 (47.2)	0 (0.0)	
Russia	0 (0.0)	1 (1.0)	
South Korea	4 (7.5)	0 (0.0)	
Missing	8 (15.1)	2 (1.9)	
Tumor grade, n (%)			0.3161
Grade 1	8 (15.1)	15 (14.6)	
Grade 2	25 (47.2)	58 (56.3)	
Grade 3	20 (37.7)	26 (25.2)	
Missing	0 (0.0)	4 (3.9)	
HBV infection, n (%)			<0.001
Yes	13 (24.5)	4 (3.9)	
No	33 (62.3)	92 (89.3)	
Missing	7 (13.2)	7 (6.8)	
HCV infection, n (%)			0.04
Yes	3 (5.7)	19 (18.4)	
No	43 (81.1)	77 (74.8)	
Missing	7 (13.2)	7 (6.8)	
Chronic liver disease, n (%)			0.20
Yes	0 (0)	3 (2.9)	
No	46 (86.8)	93 (90.3)	
Missing	7 (13.2)	7 (6.8)	
Alcohol consumption, n (%)			0.58
Yes	16 (30.2)	38 (35.9)	
No	30 (56.6)	58 (56.3)	
Missing	7 (13.2)	7 (6.8)	

Supplementary Table S2: Somatic mutation data used to generate Figure 1A with functional annotation

See Supplementary File 1

Supplementary Table S3: Mutation frequency of known HCC genes in Asians and Caucasians

Gene	Asian (n=54)	Caucasian (n=104)	P
RB1	10 (18.5)	3 (2.9)	0.001
TP53	23 (42.6)	23 (22.1)	0.01
EGF	3 (5.6)	2 (1.9)	0.34
FGF14	1 (1.9)	0 (0)	0.34
PDGFB	1 (1.9)	0 (0)	0.34
CTNNB1	12 (22.2)	30 (28.8)	0.45
HGF	0 (0)	2 (1.9)	0.55
PDGFD	0 (0)	2 (1.9)	0.55
ARID1A	6 (11.1)	8 (7.7)	0.56
ARID2	1 (1.9)	4 (3.8)	0.66
RPS6KA3	1 (1.9)	5 (4.8)	0.66
PDGFA	1 (1.9)	1 (1)	1.00
KRAS	1 (1.9)	3 (2.9)	1.00
MAPK1	0 (0)	1 (1)	1.00
MAPK13	0 (0)	1 (1)	1.00
MAPK14	0 (0)	1 (1)	1.00
MAPK4	0 (0)	1 (1)	1.00
DNAJC22	1 (1.9)	1 (1)	1.00
CYP2E1	0 (0)	1 (1)	1.00
CYP2F1	0 (0)	1 (1)	1.00

Supplementary Table S4: Differentially altered pathways/biological processes between Asian and Caucasian HCC patients in TCGA (p-value <0.01)

GO Symbol	GO Term	Proportion in Asians	Proportion in Caucasians	P
**GO pathways altered at a higher freqeuncy in Asian paitents**				
GO:0071930	negative regulation of transcription involved in G1/S transition*	0.208	0.019	1.38E-04
GO:0090230	regulation of centromere complex assembly*	0.189	0.019	3.93E-04
GO:0030512	negative regulation of transforming growth factor beta receptor signaling pathway*	0.736	0.447	6.69E-04
GO:1901673	regulation of mitotic spindle assembly	0.151	0.01	8.03E-04
GO:0000075	cell cycle checkpoint*	0.245	0.049	8.32E-04
GO:0097252	oligodendrocyte apoptotic process*	0.434	0.214	5.23E-03
GO:1902253	regulation of intrinsic apoptotic signaling pathway by p53 class mediator*	0.434	0.214	5.23E-03
GO:0034349	glial cell apoptotic process*	0.189	0.039	5.35E-03
GO:0097284	hepatocyte apoptotic process*	0.245	0.078	5.83E-03
GO:0031497	chromatin assembly*	0.472	0.243	6.21E-03
GO:0007346	regulation of mitotic cell cycle*	0.396	0.184	6.35E-03
GO:0038085	vascular endothelial growth factor binding	0.132	0.019	7.60E-03
GO:0070266	necroptotic process*	0.528	0.301	8.60E-03
GO:0090343	positive regulation of cell aging*	0.434	0.223	9.14E-03
**GO pathways altered at a higher freqeuncy in Caucasian paitents**				
GO:0008285	negative regulation of cell proliferation	0.849	0.981	2.93E-03
GO:0071236	cellular response to antibiotic	0	0.126	4.68E-03
GO:0032700	negative regulation of interleukin-17 production	0	0.117	8.62E-03

Functions marked with asterisk indicate terms which were primarily driven by TP53 or RB1

Supplementary Table S5: Associations of HCC risk factors with selected mutated genes and pathways

See Supplementary File 2
